# Stress and deformation analysis of the steel pipe arch integral lifting construction process of a continuous beam arch bridge

**DOI:** 10.1038/s41598-024-60325-x

**Published:** 2024-05-05

**Authors:** Hui Xiang, Dadong Xia, Sheng Wang, Zizheng Zhang, Shang Luo, Wei Chen

**Affiliations:** 1 China State Construction Railway Investment & Engineering Group Co., Ltd, Beijing, 102600 China; 2 School of Civil Engineering, Central South University, Changsha, 410075 China

**Keywords:** Civil engineering, Computational methods

## Abstract

The Changjinghuang Railway Xinjiang Xizhi Bridge is a (90 + 180 + 90) m continuous beam arch bridge, and the arch rib steel pipe installation adopts “short bracket assembly and overall lifting method”. In order to ensure the accuracy of closure, the stress and deformation of the arch rib and bracket must be strictly controlled. Midas Civil is used to establish the finite element model to simulate the overall lifting construction process of the arch rib. Based on the model, the stress and deformation of the arch rib and the supports are analyzed, and the determination method of the horizontal cable force under temperature variations is proposed. The results show that the stress and deformation of the arch rib and bracket meet the requirements. Considering the variation of temperature, the tension force of the horizontal cables is taken as 200 t. The construction plan proposed under the guidance of numerical calculation results has been proven by practical engineering to meet the requirement of closure accuracy, which can be used as a reference for similar projects.

## Introduction

In recent years, steel tube arch bridges have been widely used because of their high strength, small weight, strong spanning ability, and beautiful shape^1^. As bridge spans increase, the importance of arch rib construction control is becoming increasingly prominent^2^.

At present, the arch rib installation construction methods of steel pipe concrete arch bridges mainly include the bracket method, swivel method, cable hoisting method, and overall lifting method^3^. There have been many researches on the applicable scope of these construction methods. Based on a high-speed railway continuous beam arch bridge, Chen et al.^4^ made a comparative analysis of two construction schemes, the bracket method and swivel method, from the aspects of arch rib stress, construction difficulty, economy, and safety. Liu^5^, relying on the third bridge in Pingnan, Guangxi, has carried out the research on the construction control of cable hoisting cantilever assembly method for concrete filled steel tube arch bridges and proposed the “optimization algorithm of coupling influence matrix of axis and elevation”. Based on Midas Civil, Wang^6^ established the finite element model of Jihe Bridge, simulated and analyzed the stress and deformation of arch ribs and main beams in the construction process of bracket method and vertical rotation method, and compared and studied the differences of different arch forming modes. Through numerical simulation, Li et al.^7^ calculated the strength and stability of arch ribs and towers during the construction process of the swivel method and evaluated the safety of supports and local components during construction. The bracket method has the advantages of strong operability, simple construction, and easy control of the arch axis^8,9^. However, when the span and sagittal height are large, the bracket is easily deformed and difficult to hold steady. Therefore, the bracket method is mostly used in small and medium-sized bridges^10^. The swivel method is often used to construct bridges that must cross busy railways or highways because of its advantages of crossing existing traffic facilities without impact^11,12^. The cable hoisting construction method is widely used in constructing long-span arch bridges because of its strong spanning ability, heavy hoisting weight, strong environmental adaptability, and wide applicability^13,14^.

The overall lifting method uses large equipment to lift the prefabricated assembled steel arch ribs to the designed position to complete the joint^15^. The integral lifting technology was initially applied to the construction field of large and special steel structure installations. After successful application in many airports and large ports, the overall lifting method has gradually been applied to bridge construction in recent years. The Shanghe-Hangzhou High-Speed Railway Huaihe Grand Bridge adopts the method of integral lifting of large-section steel pipe arch ribs, which reduces the use of materials and shortens the construction period by one month^16^. Zizilu Bridge in Longxing New Town of Chongqing Liangjiang New District adopts the bracket method and vertical lifting method to carry out the construction of the arch rib, and the maximum closing error is 22.7 mm, meeting the design requirements^17^. In the construction of large-span arch bridges, the overall lifting method is usually used in conjunction with the bracket method. The arch ribs are assembled on the short bracket first and then lifted to the designated position. The existing engineering practice shows that the short bracket assembly and overall lifting method not only has the characteristics of simple operation and high safety of the bracket method, but also has the advantages of short construction period and less high altitude operation of the overall lifting method^18^. However, due to the strong dependence of the overall lifting construction method on lifting facilities, the overall lifting method started late, and engineering applications are not as rich as other methods^19^. Therefore, there are many problems that need to be solved during the construction process, such as the stability of the brackets and the deformation of the arch ribs.

Based on the development status and characteristics of the short bracket assembly and overall lifting method, this paper establishes a finite element model according to the construction plan of the Changjinghuang Railway Xinjiang Xizhi Bridge. Based on the established model, the stress and deformation of the arch rib and the supports are analyzed, and the determination method of the horizontal cable force under temperature variations is proposed. A detailed construction plan is put forward with the numerical calculation results as a guide.

## Project background

**Figure 1:**
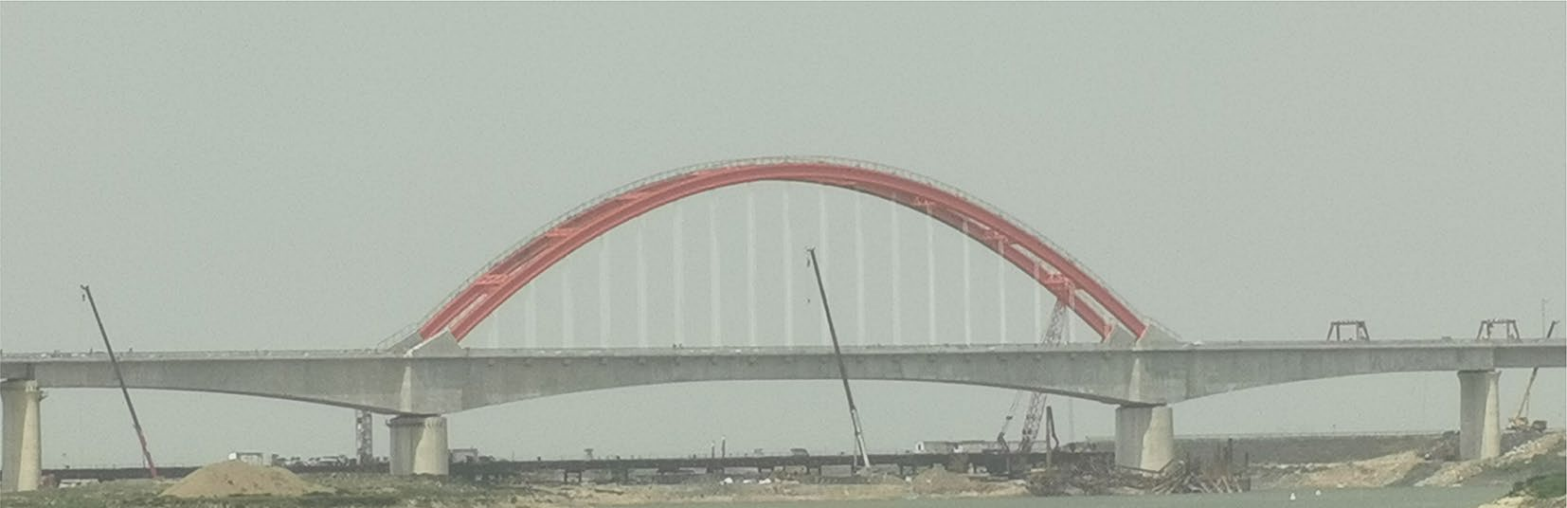
Schematic diagram of bridge structure.

The main bridge of the Changjinghuang Railway Xinjiang Bridge adopts (90 + 180 + 90) m prestressed concrete continuous girder arch structure. The schematic diagram of the bridge structure is shown in Fig. 1.

The main girder adopts a single-box double-compartment prestressed concrete box-type variable cross-section with a deck width of 13.2 m and a mid-span height of 5.0 m. The arch rib adopts vertical parallel steel pipe concrete dumbbell arch, with the arch axis adopting a quadratic parabolic line. The center distance between the two arch ribs is 11.9 m, the calculated span is 180 m, and the vector height of the arch rib elevation is 36 m. There are nine cross braces between two bays of arch ribs, and the cross braces are space truss braces. Each cross brace is composed of four main steel pipes of φ500 × 14 mm and 32 connecting steel pipes of φ250 × 10 mm. The distance between the suspenders along the bridge direction is 9 m, and there are 18 sets of double suspenders in the entire bridge. The upper end of the boom passes through the arch rib and is anchored to the tension base at the upper edge of the arch rib, while the lower end is anchored to the fixed base at the lower edge of the crossbeam at the suspension point.

The arch rib steel pipe installation adopts “short bracket assembly and overall lifting method”. The highest bracket is 17 m, and the lowest is 6.6 m. The embedded parts on the main beam are welded to the bottom of the bracket to ensure the accuracy of the support position. The arch rib assembly bracket is made of φ273 × 8 steel pipe, and the scissors support is made of〔12 a channel steel. The distribution beam at the top of the column is made of 32 a I-beam, and the web plate is welded with stiffeners corresponding to the stress-bearing position. The column of the overall lifting frame of the arch rib is made of φ426 × 8 steel pipe, and the diagonal bracing at the cantilever end of the distribution beam is made of φ273 × 12 steel pipe.

## Numerical model

**Figure 2:**
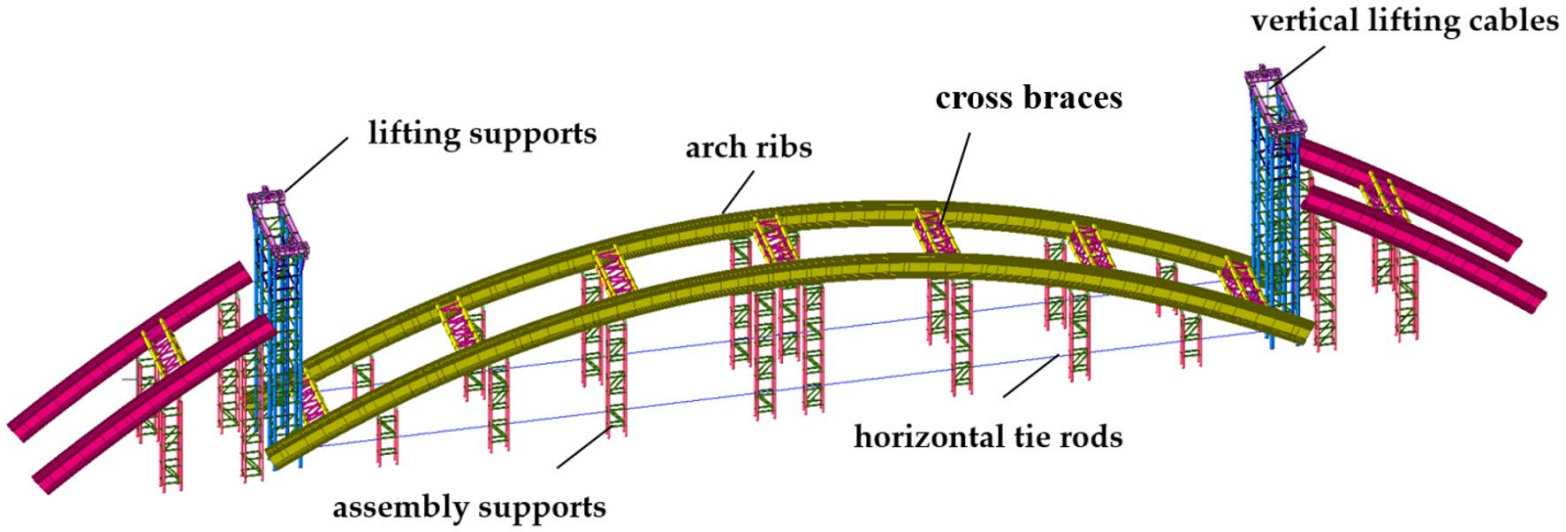
Finite element model.

Figure 2 shows the finite element model established, which mainly consists of arch ribs, assembly supports, overall lifting supports, cross braces, horizontal tie rods, and vertical lifting cables. Table 1 shows the relevant material parameters.

**Table 1:** Parameters of the superstructure and temporary support system.

Components	Elastic modulus (MPa)	Density (${\text{kg}}\,{\text{m}}^{ - 3}$)	Poisson’s ratio
Arch ribs	210,000	7850	0.3
Cross braces	210,000	7850	0.3
Supports	210,000	7850	0.3
Horizontal tie rods	195,000	8000	0.3
Vertical lifting cables	195,000	8000	0.3

The height of the lifting bracket is 28.5 m, the maximum height of the assembly bracket is 17 m, and the minimum is 6 m. The length of the lifting section of the arch rib is 112.4 m and the lifting height is 17.76 m. The arch ribs, cross braces, and supports are simulated by beam units, and the horizontal tie rods and vertical lifting cables are simulated by tension-only truss units. The tension force of the horizontal ties rods and the lifting force of the vertical lifting cables are applied by initial tension loads.

The bottom of the supports is fixed and restrained. The constraints between the assembly support, and the arch ribs are simulated by ‘Rigid connection’ in the low-level assembly stage, while ‘Only-compression elastic connection’ in the trial lifting stage. The constraints between arch ribs and cross braces are simulated by ‘Elastic connection’. The connections between the arch rib and horizontal tie rods, as well as the vertical lifting cables, all adopt a ‘Rigid connection’. The ‘Rigid connections’ are used to simulate the constraints between the vertical lifting cables and the distribution beams at the top of the overall lifting supports^20^.

Arch ribs and supports are mainly subjected to self-weight loads and wind loads. The self-weight load is automatically calculated by Midas Civil software, and the wind load is calculated by the following formula^21^:

1 $$W={K}_{1}\times {K}_{2}\times {K}_{3}\times {W}_{0}$$

where $W$: is the wind strength (Pa), ${K}_{1}$: is the shape coefficient of wind load, ${K}_{2}$: is the coefficient of wind pressure variation with height, ${K}_{3}$: is the geographical coefficient, ${W}_{0}$: is the basic wind strength (Pa).

After calculation, the wind strength of the arch rib is taken as 753.5 Pa, and the wind strength of the supports is taken as 548 Pa.

**Figure 3:**
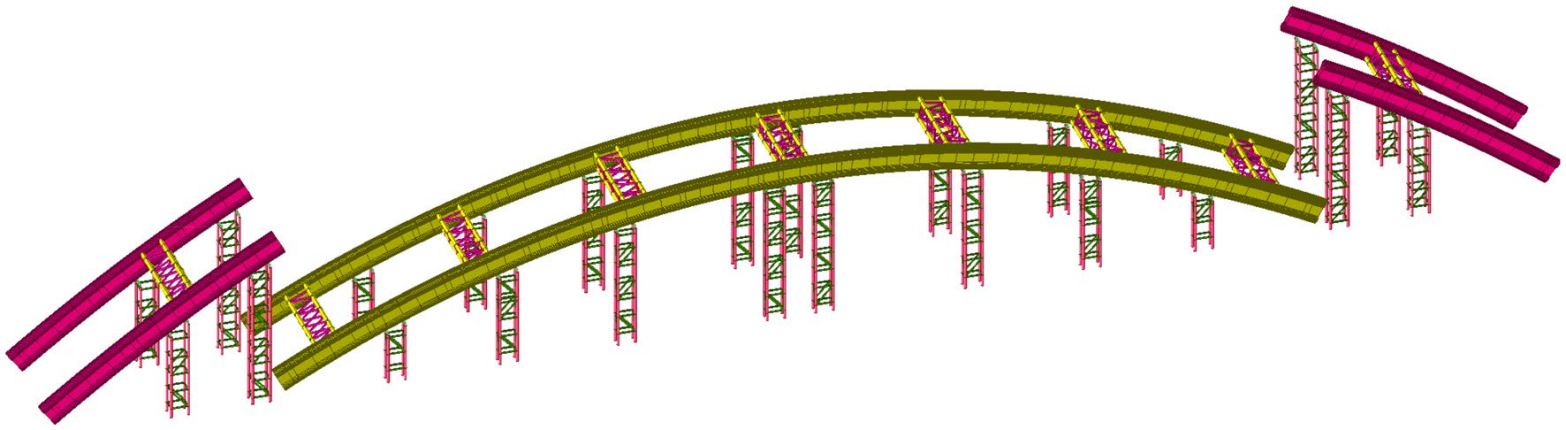
Calculation model of the low-level assembly.

**Figure 4:**
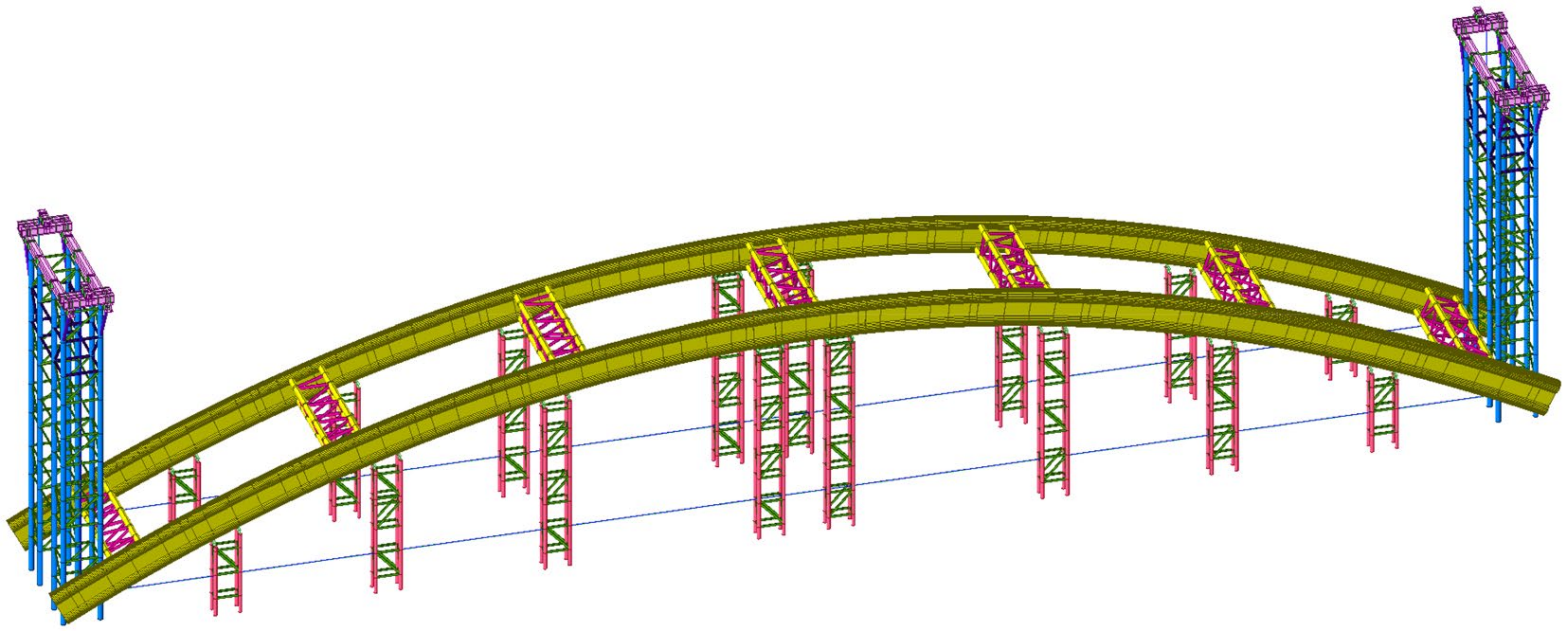
Calculation model of trial lifting.

**Figure 5:**
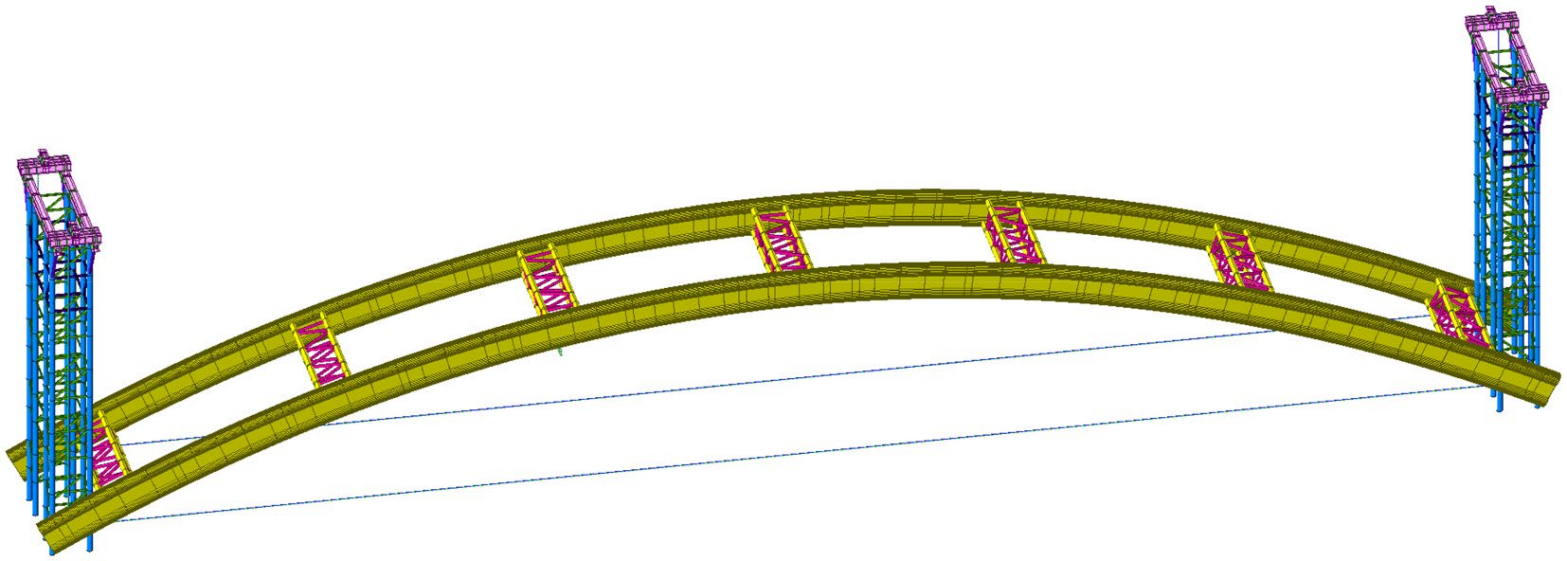
Calculation model of formal lifting.

Three construction stages, including low-level assembly, trial lifting, and formal lifting, are selected for modeling analysis to analyse the stress and deformation of the structure during the construction process. The calculation models of the three construction stages are shown in Figs. 3, 4, 5.

## Force analysis of the supports

The self-weight load of the arch rib is borne by the assembly supports in the low-level assembly stage, which is the most unfavorable working condition of the assembly supports. Therefore, the low-level assembly stage is chosen to analyze the stability of the assembly supports. The stress and deformation of the assembly support in the low-level assembly stage are shown in Fig. 7.

**Figure 6:**
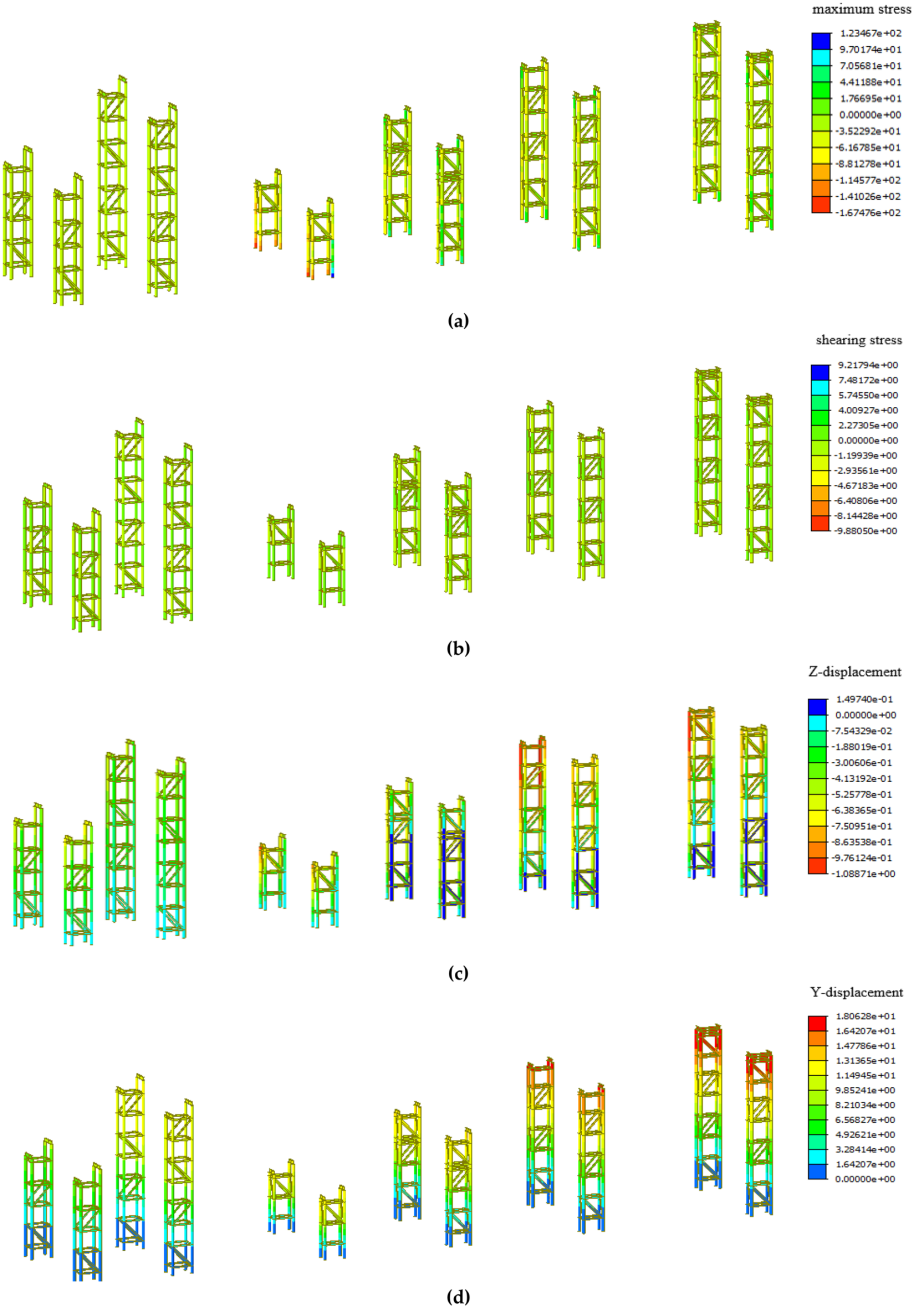
Cloud diagram of assembly supports: (**a**) Maximum stress; (Unit: MPa). (**b**) Shearing stress; (Unit: MPa). (**c**) Vertical displacement; (Unit: mm). (**d**) Horizontal displacement; (Unit: mm).

According to the standard for design of steel structures, the design value of compressive strength of Q235 steel f_d_ is 215 MPa, and the design value of shear strength f_v_ is 125 MPa^22^. As shown in Fig. 6, the maximum stress of the assembly supports is ${\upsigma }_{{{\text{max}}}} {\text{ = 167 MPa}}\,{\text{ < f}}_{{\text{d}}} {\text{ = 215 MPa}}$ and the maximum shearing stress of the assembly ssupports${\upsigma }_{{{\text{max}}}} { = 9}{\text{.8 MPa}}\,{\text{ < f}}_{{\text{v}}} {\text{ = 125 MPa}}$, which means that the strength of the assembly supports meets the design requirements. In the low-level assembly stage, both of the maximum stress and maximum shearing stress appear at the outermost assembly supports of the lifting section.

The steel structure is permitted to deflect up to 1/400 of its span length under the constant and live loads^22^. The allowable deflection of the supporting beams on the top of the assembly supports is 1000/400 = 2.5 mm, and the allowable value of horizontal displacement at the top of the support is 16,600/400 = 41.5 mm. It can be seen from Fig. 6 that the maximum vertical displacement of the assembly supports is 1 mm, appearing at the supporting beam, and the maximum horizontal displacement of the assembly supports is 18 mm, appearing at the top of the middle assembly supports of the lifting section. Therefore, the stiffness of the assembly supports meets the design requirements.

**Figure 7:**
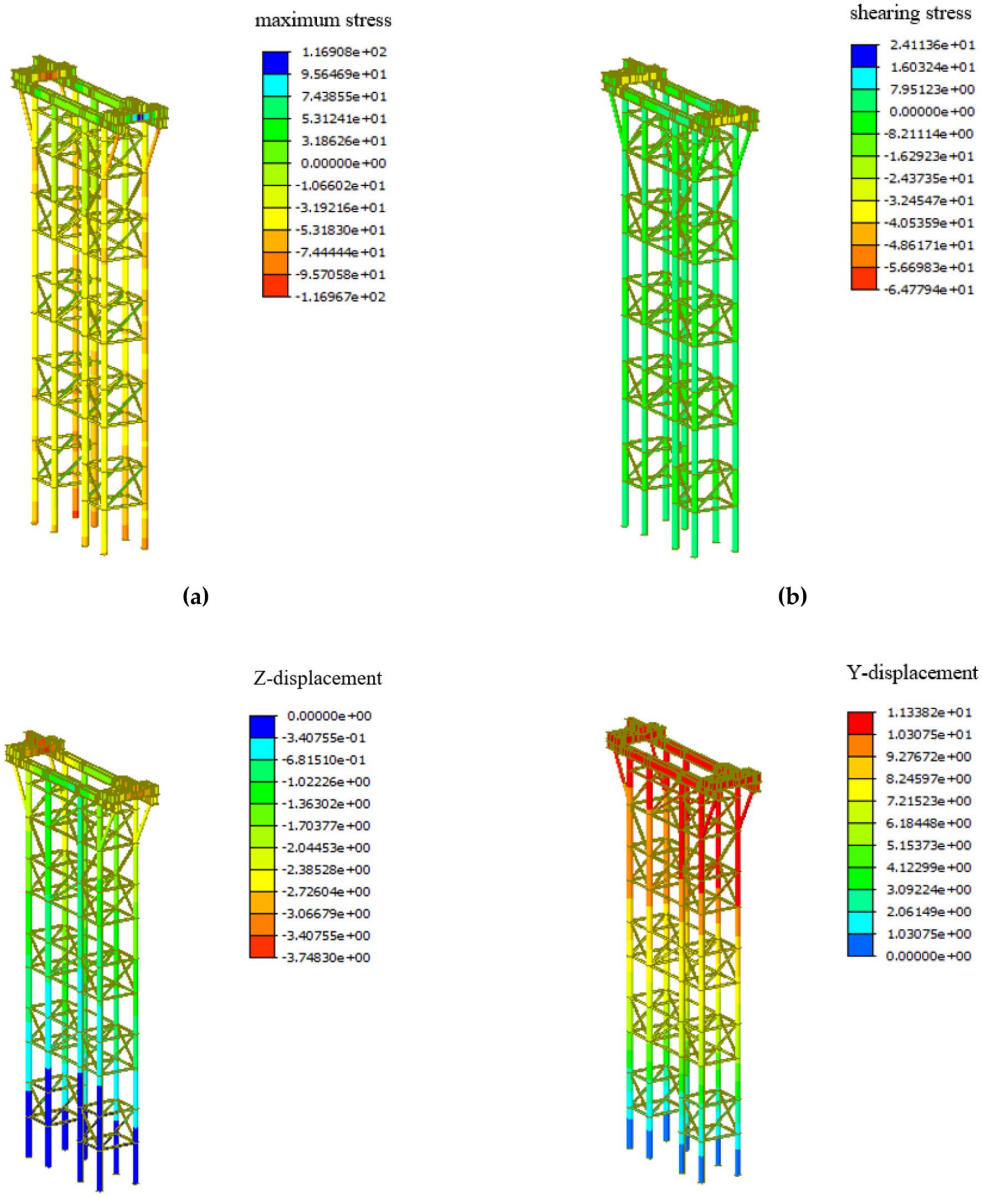
Cloud diagram of overall lifting supports: (**a**) Maximum stress; (Unit: MPa) (**b**) Shearing stress; (Unit: MPa) (**c**) Vertical displacement; (Unit: mm) (**d**) Horizontal displacement; (Unit: mm).

The self-weight load of the arch rib in the formal lifting stage is borne by the overall lifting supports, which is the most unfavorable working condition of the overall lifting supports. Therefore, the overall lifting stage is chosen to analyse the stability of the lifting supports.The stress and deformation of the overall lifting supports in the low-level assembly stage are shown in Fig. 7.

As shown in Fig. 7, the maximum stress of the overall lifting supports is ${\upsigma }_{{{\text{max}}}} {\text{ = 116 MPa}}\,{\text{ < f}}_{{\text{d}}} {\text{ = 215 MPa}}$ and the maximum shearing stress of the overall lifting spports is ${\upsigma }_{{{\text{max}}}} {\text{ = 64MPa}}\,{\text{ < f}}_{{\text{v}}} {\text{ = 125 MPa}}$, which means that the strength of the overall lifting supports meets the design requirements. In the formal lifting stage, the maximum stress appears at the bottom of the overall lifting supports, and the maximum shearing stress appears at the supporting beam on the top of the overall lifting supports.

The allowable deflection of the supporting beams on the top of the overall lifting supports is 2000/400 = 5 mm, and the allowable value of horizontal displacement at the top of the support is 28,000/400 = 70 mm. It can be seen from Fig. 7, the maximum vertical displacement of the overall lifting supports is 3.7 mm appearing at the supporting beam, and the maximum horizontal displacement of the overall lifting supports is 11 mm appearing at the top of the overall lifting supports. Therefore, the stiffness of the overall lifting supports meets the design requirements.

## Displacement and stress of the arch ribs

### Determination of the optimal horizontal cable force

**Figure 8:**
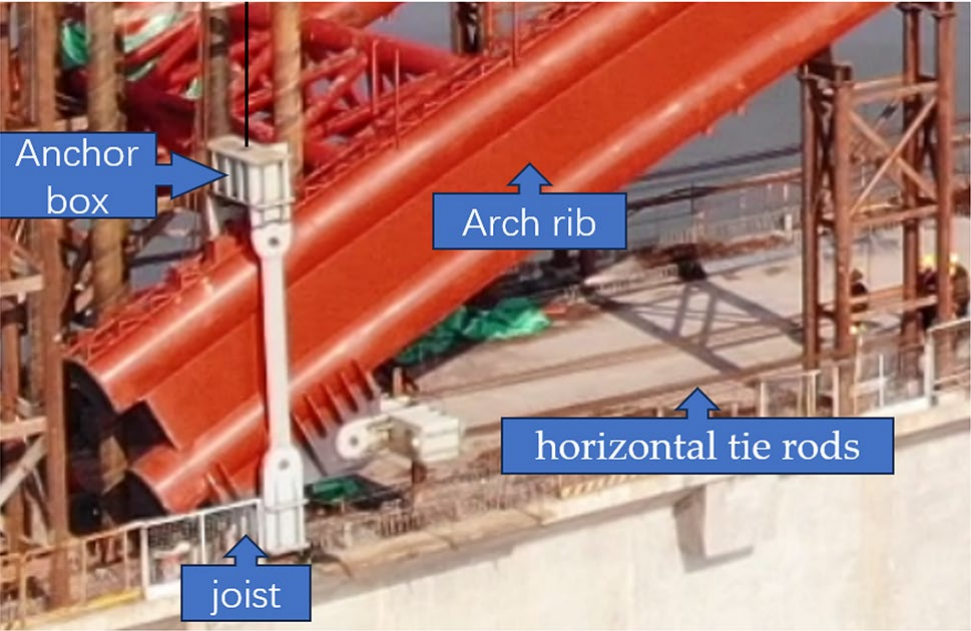
Lifting devices.

As shown in Fig. 8, the lifting device consists of anchor box,joist,horizontal tie rods and vertical lifting cables. The anchor box is connected with the vertical lifting cables, and the joist is connected with the horizontal tie rods. The anchor box and joist are used to fix the arch rib. The vertical lifting cables and the numerical control jack realize the lifting function.

During the integral lifting of the arch rib, horizontal tie rods are arranged at both ends of each arch rib to constrain the horizontal deformation of the ends. Meanwhile, vertical lifting cables are installed at both ends of each arch rib to provide vertical support for the arch rib. Therefore, the overall stress state of the arch rib during the integral lifting process is that the arch rib forms a self-balancing state under the combined action of the self-weight, the horizontal tensile force, and the vertical lifting force. By applying accurate horizontal tension cable forces and adopting reasonable construction measures and monitoring methods, the control requirements for the integral lifting of the arch rib can be achieved. To obtain the reasonable cable force, the following methods are employed in the formal lifting calculation model:

① Set the stiffness of the horizontal cables in the formal lifting calculation model to infinite, so that the cables are passively stressed, and run the model to obtain the initial cable force Ti.
② Fine-tune the initial cable force Ti to make the horizontal displacement at both ends of the arch rib as close to 0 as possible, and obtain the optimal cable force To.
③ Change the temperature, repeat the above steps, and obtain the optimal cable force To under each temperature condition.

**Figure 9:**
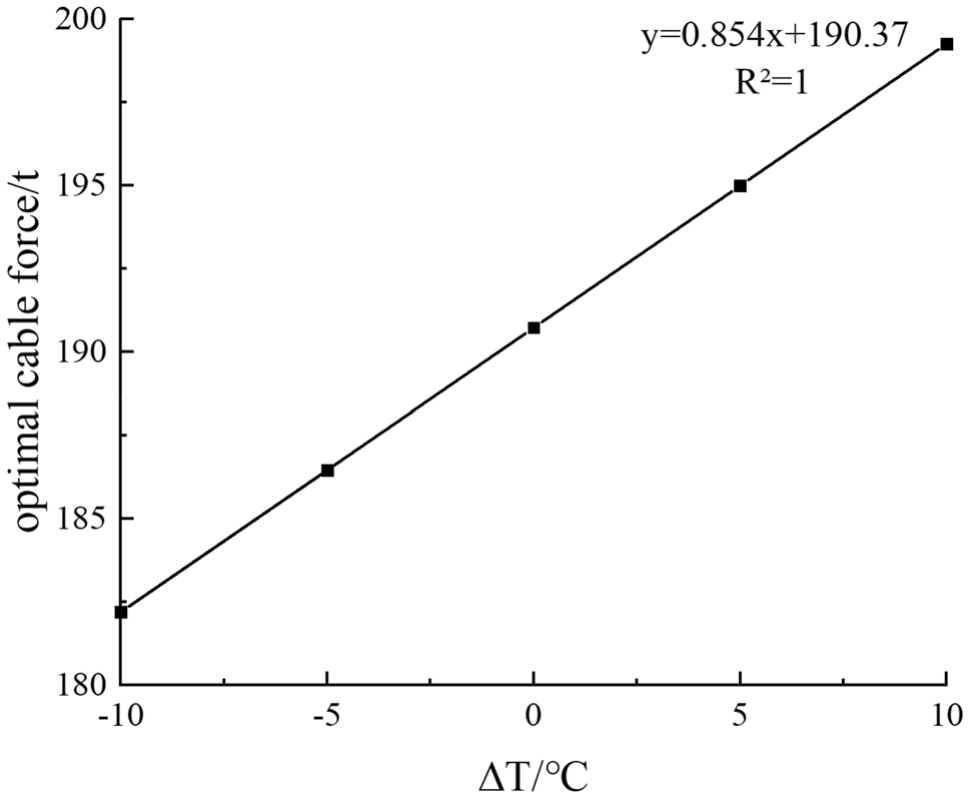
Optimal cable force in different temperature.

After calculation, the initial cable force value Ti was determined to be 190.05 t, with a horizontal displacement of 0.54 mm at both ends of the arch rib. After continuous adjustments, the optimal cable force value T0 reached 190.73 t, resulting in a horizontal displacement of 0.01 mm at both ends of the arch rib. We then calculated the optimal cable force for temperature variations ranging from ΔT = − 10–10 °C and performed a linear fitting. The results are presented in Fig. 9. It can be seen from the results presented in Fig. 9 that the optimal horizontal cable force is linearly correlated with the overall temperature of the arch rib. Considering the temperature of the construction site, the tension force of the horizontal tie is taken as 200 t.

### Force and deformation analysis of the lifting process

The self-weight of the lifting section of the arch rib is about 440 t, and the tension force of the horizontal tie rods is 200 t. Due to the large lifting weight and tension force, the horizontal force and lifting force should be loaded in a graded and synchronized manner to keep the stress and deformation of the arch rib and brackets under control. As shown in Table 2, the tension in the horizontal and vertical cables is increased by 25% of the target cable force each time until the arch ribs detach from the supports, corresponding to CS1 to CS5, respectively. The trial lifting loading process is divided into five working conditions, and the specific loading conditions are shown in Table 2.

**Table 2:** Trial lifting stage loading conditions.

Component	Target force (t)	CS1	CS2	CS3	CS4	CS5
Horizontal tie rods (%)	200	25%	50%	75%	100%	Detach
Vertical lifting cables (%)	440	25%	50%	75%	100%	

**Figure 10:**
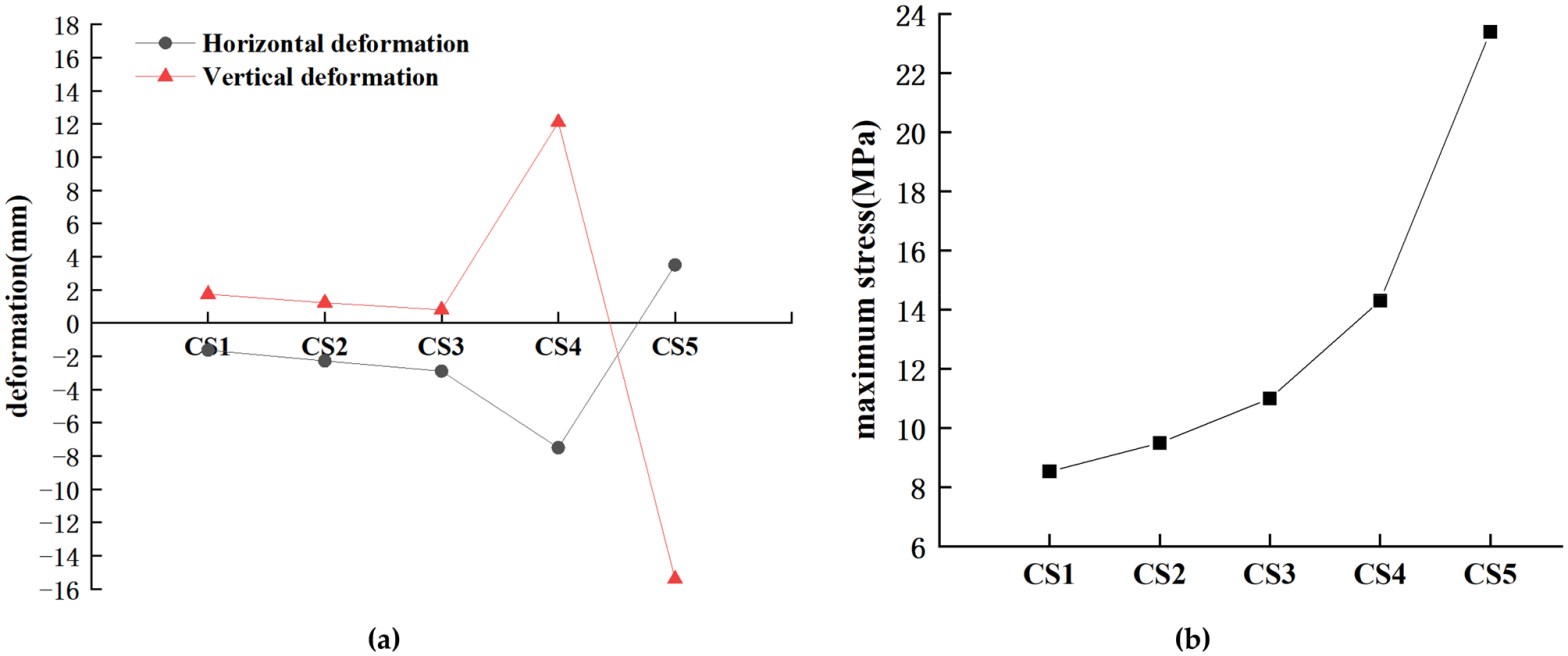
Arch rib deformation and stress results: (**a**) Horizontal and vertical deformation; (Unit: MPa). (**b**) Maximum stress; (Unit: MPa).

Figure 10 shows the deformation and stress of the arch rib after the tension force is applied in each stage of CS1–CS5. In CS1–CS3, as the load increases, the maximum stress of the arch rib increases linearly, while the vertical elongation deformation and horizontal shrinkage deformation of the arch rib do not change much. This is because the arch ribs in CS1–CS3 have not been completely detached, which leads to a certain supporting effect on the arch ribs, so the deformation and stress of the arch ribs are more reasonable. In CS3–CS4, the horizontal deformation and vertical deformation of the arch rib increase greatly because the middle part of the arch rib is decoupled from the supports when the horizontal force and vertical force reach the target value. In CS4–CS5, due to the detachment of the arch ribs, the vault is deflected downward by its own weight, so the vertical deformation of the arch ribs changes from elongation deformation to shrinkage deformation, and the horizontal deformation of the arch ribs is on the contrary. Although CS5 has larger deformation and stress than the previous four loading conditions, the mechanical performance of the arch rib structure during the lifting process is still good, which can meet the design and construction requirements.

## Short bracket assembly and overall lifting method

The construction process of short bracket assembly and the overall lifting method is as follows: supports installation → low-level assembly → trial lifting → formal lifting → arch rib closing

### Supports installation

The assembly supports are installed from the arch foot to the middle of the span, and the specific construction steps are as follows: reviewing the position of the pre-embedded parts → assembling the pipe piles on site → assembling the connection system → installing the lattice columns → installing the transverse distributing beams → inspecting and checking the acceptance.

The distribution beam on the top of the column adopts 40 a I-beam, and the I-beam web plate is welded with stiffening ribs corresponding to the stress position. The top of the distribution beam adopts a steel pipe short pier as the elevation adjustment pier, and the angle is set according to the arch axis line type and welded on the distribution beam. To maintain the lateral stability of assembly supports, pre-embedded parts are welded with steel pipes at the bottom of the support. When the support exceeds 12 m, wind cables are installed. The top of the wind cables is connected to the top of the support, and the bottom is connected to the pre-embedded reinforcement on the beam surface. After the bracket installation is completed, the welds and connections of the bracket are checked, and the position of the arch rib positioning saddle is adjusted so that the axis and elevation of the positioning saddle meet the installation requirements.

### Low-level assembly

**Figure 11:**
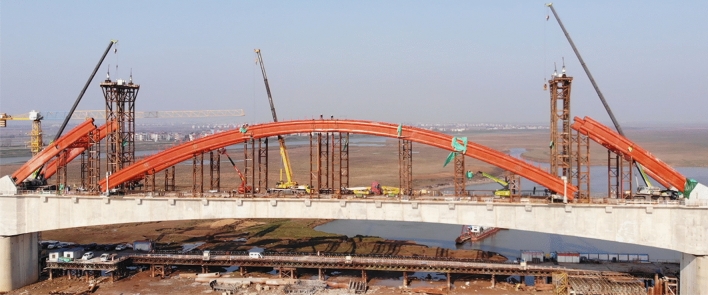
Construction of the low-level assembly.

There are 4 small segments in the arch rib of the non-lifting section, and the weight of a single piece is about 20.6 t. They are all installed separately with 80t truck cranes, and the lifting height is 13.5 m. The next construction section can only be carried out after the temporary plate is firmly welded. After the left and right arch rib segments on one side are installed, hoist and install the cross braces in time and weld them firmly with the arch rib segments on both sides. The arch rib of the lifting section is divided into 9 sections, respectively installed with two 80 t truck-mounted floating cranes. The weight of a single piece is about 20.7 t, and the lifting height is 20 m. The cross braces must be installed timely to increase the lateral overturning resistance of the installed arch. Fig. 11 shows the complete diagram of low-level assembly.

### Trial lifting

After completing the preparatory work for the trial lifting, carry out the trial lifting work according to the following steps. Step 1: Release all consolidation between the arch rib and the assembly supports, and the release sequence is carried out from both sides to the middle of the span. Step 2: According to 25%, 50%, 75%, and 100% of the target values of the horizontal and vertical tension, the horizontal tie rods and vertical lifting cables are gradually and symmetrically stretched until the arch rib is basically separated from the assembly supports. Step 3: Check whether the weld seam of the structure is normal, and whether the deformation of the structure is within the allowable range. After meeting the design requirements, slowly lift the arch rib. When the minimum distance between the arch rib and the support is 200 mm, let the arch rib stand still to complete the trial lifting work.

### Formal lifting

**Figure 12:**
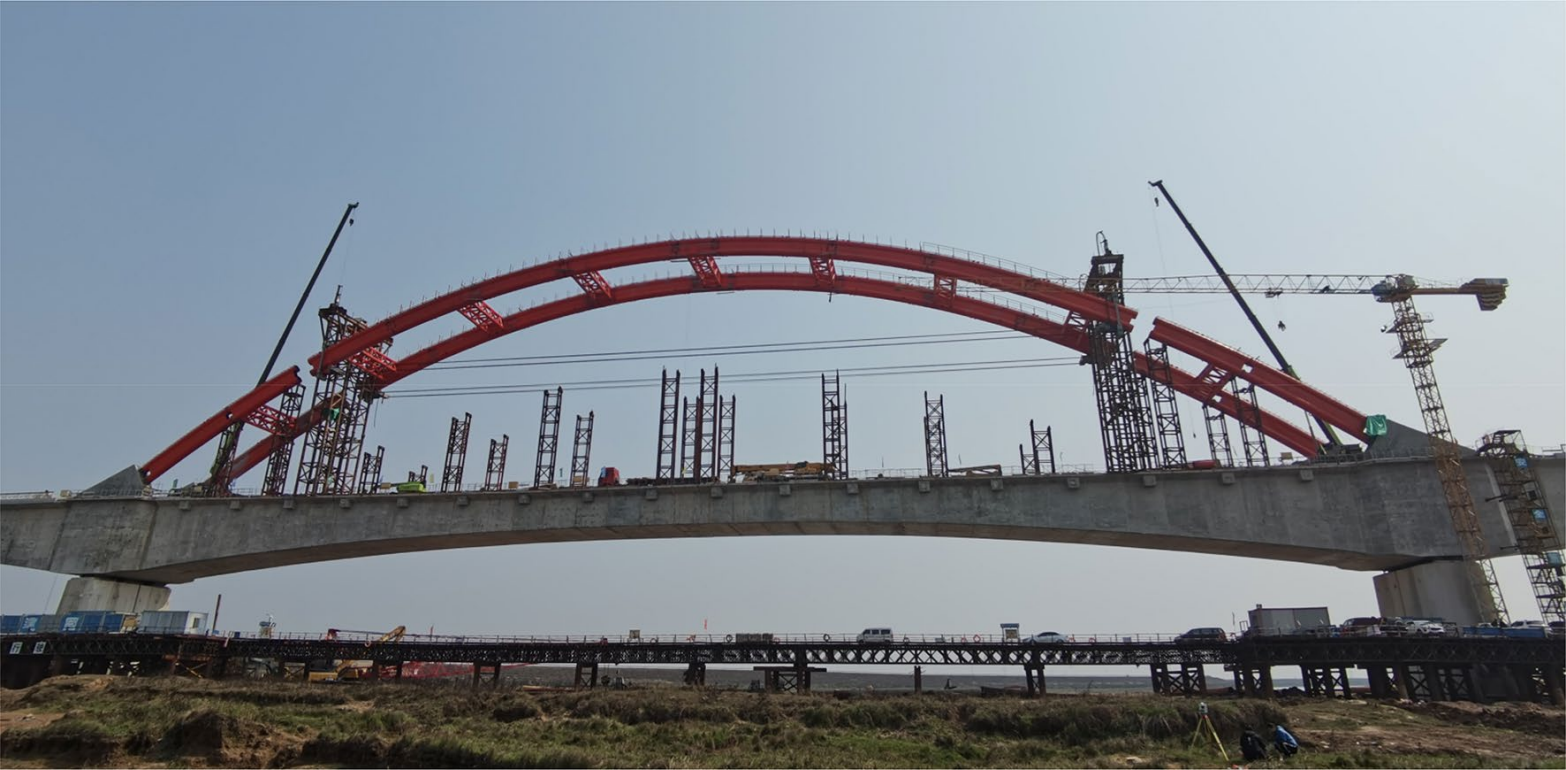
Construction of formal lifting.

When there is no abnormality in the lifting cables, overall lifting supports, and lifting equipment system, and the weather is suitable, the official lifting work will start. Four 300t lifting jacks work simultaneously, and the lifting speed is 4–6 m/h. During the lifting process, the synchronization of the four arch feet of the arch rib should be remeasured in time. When the four arch feet are not in the same horizontal plane, the jack should be manually controlled to adjust its position. When the arch rib is raised to 200 mm below the design position, the lifting speed should be slowed down, and measurements and accurate positioning should be made to ensure that the final elevation of the arch rib meets the design requirements. When the arch rib is lifted to the design height, retest whether the chord length of the arch rib changes. If there is a change, the arch rib can be fine-tuned by regulating the tension of the horizontal tie rods. The formal lifting is completed as shown in Fig. 12.

### Arch rib closing

Temporary locking is performed in the early morning when temperatures are cool and steady. After measuring the length of the merging sections, the merging sections are cut precisely and finally lifted and welded. As the installation of the merging segments plays a key role in the final shape of the arch, human errors should be eliminated as much as possible during the operation. When welding of the 4 joint segments of the arch rib is completed, unload the horizontal tie rods first, and then unload the vertical lifting cables.

## Conclusions

Through the analysis of the stability and stress of the supports and arch ribs and the detailed presentation of the on-site construction cases, the following conclusions are obtained:

1. The stress and deformation of the assembly support and overall lifting supports meet the design requirements, and the structure is safe in each construction stage.
2. In the low-level assembly stage, the maximum stress is 167 MPa, appearing at the bottom of the outermost assembly supports of the lifting section, and the maximum vertical displacement is 1 mm, appearing at the supporting beam.
3. In the formal lifting stage, the maximum stress is 116 MPa, appearing at the bottom of the overall lifting supports, and the maximum vertical displacement of the overall lifting supports is 3.7 mm, appearing at the supporting beam.
4. The determination method of the horizontal cable force proposed in this paper can quickly determine the optimal cable force values at different temperatures. The result shows the optimal horizontal cable force is linearly correlated with the overall temperature of the arch rib.

## Data Availability

All data generated or analysed during this study are included in this published article.
